# Unified and Exact Framework for Variance-Based Uncertainty Relations

**DOI:** 10.1038/s41598-019-56803-2

**Published:** 2020-01-10

**Authors:** Xiao Zheng, Shao-Qiang Ma, Guo-Feng Zhang, Heng Fan, Wu-Ming Liu

**Affiliations:** 1 School of Physics, Beihang University, Xueyuan Road No. 37, Beijing, 100191 China; 2 Beijing National Laboratory for Condensed Matter Physics, Institute of Physics, Chinese Academy of Sciences, Beijing, 100190 China; 3 School of Physical Sciences, University of Chinese Academy of Sciences, Beijing, 100190 China; 4 CAS Central of Excellence in Topological Quantum Computation, Beijing, 100190 China; 5 Songshan Lake Materials Laboratory, Dongguan, Guangdong, 523808 China

**Keywords:** Quantum information, Quantum mechanics

## Abstract

We provide a unified and exact framework for the variance-based uncertainty relations. This unified framework not only recovers some well-known previous uncertainty relations, but also fixes the deficiencies of them. Utilizing the unified framework, we can construct the new uncertainty relations in both product and sum form for two and more incompatible observables with any tightness we require. Moreover, one can even construct uncertainty equalities to exactly express the uncertainty relation by the unified framework, and the framework is therefore exact in describing the uncertainty relation. Some applications have been provided to illustrate the importance of this unified and exact framework. Also, we show that the contradiction between uncertainty relation and non-Hermitian operator, i.e., most of uncertainty relations will be violated when applied to non-Hermitian operators, can be fixed by this unified and exact framework.

## Introduction

Quantum uncertainty relations^1–3^, expressing the impossibility of the joint sharp preparation of the incompatible observables^4,5^, are the most fundamental differences between quantum and classical mechanics^6–9^. The uncertainty relation has been widely used in the quantum information science^10,11^, such as quantum non-cloning theorem^12,13^, quantum cryptography^14–17^, entanglement detection^18–22^, quantum spins squeezing^23–26^, quantum metrology^27–29^, quantum synchronization^30,31^ and mixedness detection^32,33^. In general, the improvement in uncertainty relations will greatly promote the development of quantum information science^18,28,34–36^.

The variance-based uncertainty relations for two incompatible observables *A* and *B* can be divided into two forms: the product form Δ*A*^2^Δ*B*^2^ ≥ *LB*_*p*_^2,3,5,37,38^ and the sum form Δ*A*^2^ + Δ*B*^2^ ≥ *LB*_*s*_^39–42^, where *LB*_*p*_ and *LB*_*s*_ represent the lower bounds of the two forms uncertainty relations, and Δ*Q*^2^ is the variance of *Q* (To make sure that the quantity measuring the uncertainty will be a real number, the variance is taken as 〈(*Q* − 〈*Q*〉)^†^(*Q* − 〈*Q*〉) for non-Hermitian operators. Here the 〈*Q*〉 represents the expected value of *Q*). The product form uncertainty relation cannot fully capture the concept of the incompatible observables, because it can be trivial; i.e., the lower bound *LB*_*p*_ can be null even for incompatible observables^39,40,43,44^. This deficiency is referred to as the triviality problem of the product form uncertainty relation. In order to fix the triviality problem, Maccone and Pati deduced a sum form uncertainty relation with a non-zero lower bound for incompatible observables^44^, firstly showing that the triviality problem can be addressed by the sum form uncertainty relation. Thus, the sum form uncertainty relations were considered to be stronger than the product form uncertainty relations, and since then, lots of effort has been made to investigate the uncertainty relation in the sum form^18,39,45–48^. However, most of the sum form uncertainty relations depend on the orthogonal state to the state of the system, and thus are difficult to apply to a high dimension Hilbert space^39^. There also exist the uncertainty relations based on the entropy^6,7,12,49^ and skew information^50^, which may not suffer the triviality problem, but they cannot capture the incompatibility in terms of the experimentally measured error bars, namely variances^9,44^.

Here we only focus on the uncertainty relation based on the variance. Despite the significant progress on this subject, previous works mainly study the variance-based uncertainty relations, *separately*. A natural question is raised: can these various uncertainty relations be integrated into a unified framework? If so, can the unified framework fix the deficiencies in the previous uncertainty relations and provide a more accurate description for quantum uncertainty relation? In other words, can the unified framework provide a stronger theoretical system for quantum uncertainty relation?

**Figure 1:**
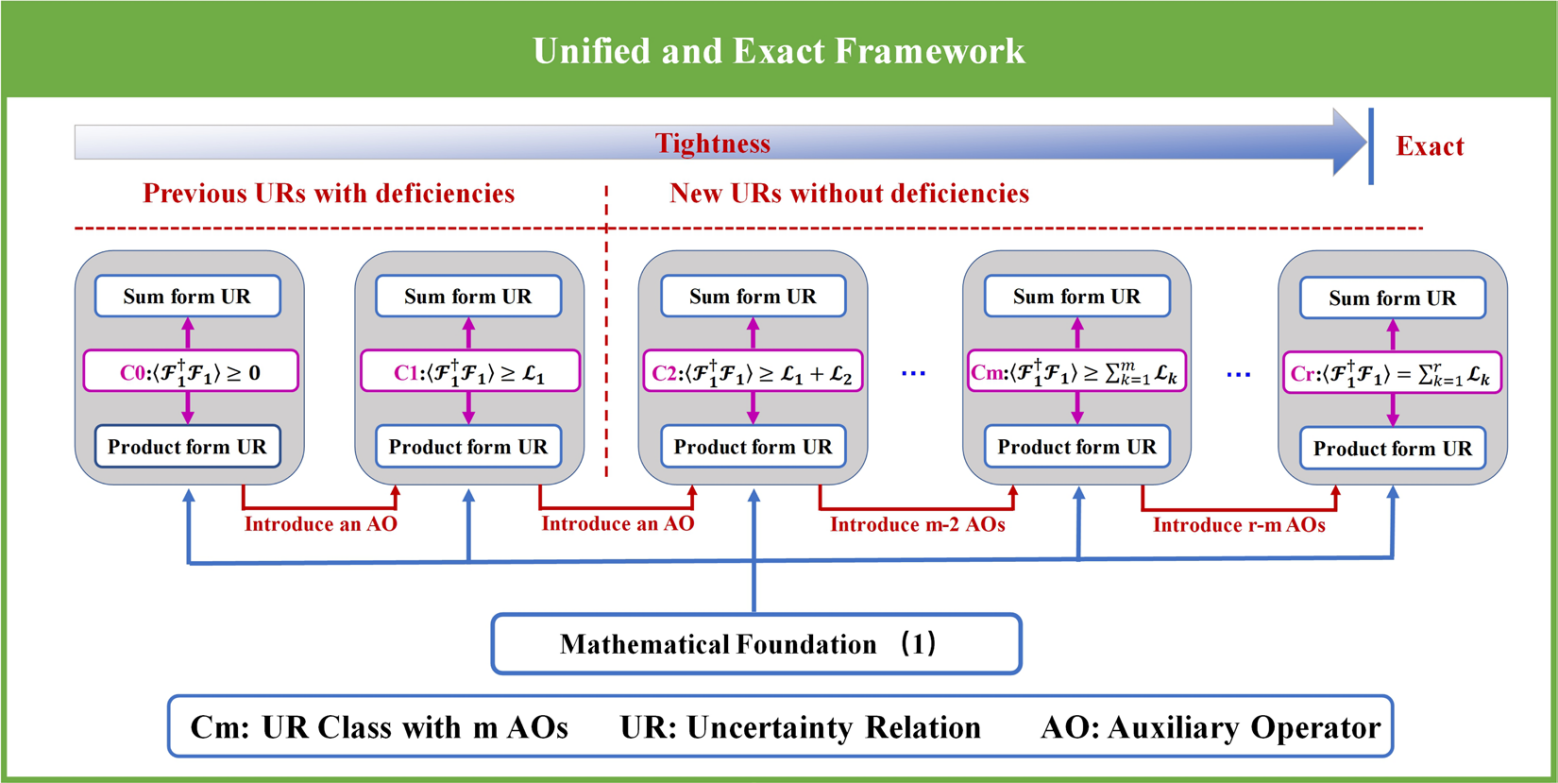
Illustration of the unified and exact framework. Utilizing Eq. (1), we construct the unified and exact framework for uncertainty relations. The uncertainty relations in the framework can be uniformly obtained by means of introducing auxiliary operators, and they are divided into different classes according to the number of auxiliary operators involved. We denote the class corresponding to *m* auxiliary operators by Class-Cm. The Class-Cm contains lots of uncertainty relations in both product form and sum form for two and more incompatible observables, and these uncertainty relations can be uniformly expressed by a general formula, namely $\langle { {\mathcal F} }_{1}^{\dagger }{ {\mathcal F} }_{1}\rangle \ge \mathop{\sum }\limits_{k=1}^{m}{ {\mathcal F} }_{k}$ with ${ {\mathcal L} }_{k}=(|\langle {[{ {\mathcal F} }_{k},{{\mathscr{O}}}_{k}]}_{{\mathscr{G}}}\rangle {|}^{2}+|\langle {\{{ {\mathcal F} }_{k},{{\mathscr{O}}}_{k}\}}_{{\mathscr{G}}}{\rangle |}^{2})/4|\langle {{\mathscr{O}}}_{k}^{\dagger }{{\mathscr{O}}}_{k}\rangle |$ and ${{\mathscr{O}}}_{k}$ being an arbitrary auxiliary operator. Some well-known previous uncertainty relations actually belong to the weakest two classes and the deficiencies of them are the common characteristics of the classes they belong to. The deficiencies can be completely fixed by uncertainty relations in the stronger classes. The uncertainty relations in the stronger class in general possess the tighter lower bound, because they involve more auxiliary operators. The uncertainty relation can be exactly expressed by equalities when *r* auxiliary operators, which satisfy a given condition, are introduced, and these equalities constitute the exact Class-Cr. Remarkably, there exists no limitation on the choice of the auxiliary operator when the uncertainty relation are not required to be exactly expressed.

To provide such a unified framework, we construct an equality in terms of second-order origin moment, and introduce a new concept of “auxiliary operator”, which is used to make uncertainty relation be expressed more accurately. Utilizing this equality, we mathematically construct several different and inequivalent uncertainty relation classes, and each class contains lots of uncertainty relations in both sum form and product form for two and more incompatible observables. These uncertainty relations include both well-known previous uncertainty relations and new stronger uncertainty relations. In physics, the uncertainty relations in these different classes can all be obtained by introducing auxiliary operators, and the reason why these uncertainty relations are classified into different classes is that the number of the auxiliary operators introduced is different. That is to say, these uncertainty relations can be uniformly deduced, described and classified by the auxiliary operator, and thus we provide a unified framework for uncertainty relations, as shown in Fig. 1. Also, we deduce that the uncertainty inequality will become uncertainty equality when a suit number of auxiliary operators are introduced, thus the unified framework is exact in describing uncertainty relation.

The paper is organized as follows. Sec. 2 is to construct the mathematical foundation of this unified framework, namely the equality based on the second-order origin moment. The uncertainty relation classes in the unified framework, so do the uncertainty relations in these classes, can all be mathematically deduced from the equality, and we thus refer to the equality as the mathematical foundation of the unified and exact framework. In Sec. 3, we construct a unified and exact framework for uncertainty relations. An application in non-Markovianity witness has been provided to illustrate the importance of the unified and exact framework in Sec. 4. Sec. 5 is provided to discuss the applicability of this unified and exact framework to non-Hermitian operators. The conclusion and discussion are presented in Sec. 6.

## Mathematical Foundation

As mentioned above, the equality based on the second-order origin moment provides a mathematical foundation for the unified framework. Thus, before constructing the unified framework, we should firstly introduce this equality, which reads (for more detail, please see Appendix A):

1 $$\langle {{\mathscr{A}}}^{\dagger }{\mathscr{A}}\rangle \langle { {\mathcal B} }^{\dagger } {\mathcal B} \rangle =\frac{|\langle {[{\mathscr{A}}, {\mathcal B} ]}_{{\mathscr{G}}}\rangle {|}^{2}}{4}+\frac{|\langle {\{{\mathscr{A}}, {\mathcal B} \}}_{{\mathscr{G}}}\rangle {|}^{2}}{4}+\langle {{\mathscr{C}}}^{\dagger }{\mathscr{C}}\rangle \langle { {\mathcal B} }^{\dagger } {\mathcal B} \rangle ,$$

where ${\mathscr{A}}$ and ${\mathcal B}$ represent two arbitrary operators, the remainder $\langle {{\mathscr{C}}}^{\dagger }{\mathscr{C}}\rangle \langle { {\mathcal B} }^{\dagger } {\mathcal B} \rangle \ge 0$ with ${\mathscr{C}}={\mathscr{A}}-\langle { {\mathcal B} }^{\dagger }{\mathscr{A}}\rangle  {\mathcal B} /\langle { {\mathcal B} }^{\dagger } {\mathcal B} \rangle$, and $\langle {{\mathscr{Q}}}^{\dagger }{\mathscr{Q}}\rangle$ is the second-order origin moment of the operator ${\mathscr{Q}}$. ${[{\mathscr{A}}, {\mathcal B} ]}_{{\mathscr{G}}}$ and ${\{{\mathscr{A}}, {\mathcal B} \}}_{{\mathscr{G}}}$ are the non-Hermitian extension of the commutator and anti-commutator, which are therefore named as the generalized commutator and generalized anti-commutator, respectively. They are defined as:

2 $${[{\mathscr{A}}, {\mathcal B} ]}_{{\mathscr{G}}}={{\mathscr{A}}}^{\dagger } {\mathcal B} -{ {\mathcal B} }^{\dagger }{\mathscr{A}},\,{\{{\mathscr{A}}, {\mathcal B} \}}_{{\mathscr{G}}}={{\mathscr{A}}}^{\dagger } {\mathcal B} +{ {\mathcal B} }^{\dagger }{\mathscr{A}}.$$

The generalized commutator and anti-commutator will reduce to the normal ones when ${\mathscr{A}}$ and ${\mathcal B}$ are both Hermitian. We say that ${\mathscr{A}}$ and ${\mathcal B}$ are generalized incompatible (generalized anti-incompatible) with each other hereafter when ${[{\mathscr{A}}, {\mathcal B} ]}_{{\mathscr{G}}}\ne 0$ $({\{{\mathscr{A}}, {\mathcal B} \}}_{{\mathscr{G}}}\ne 0)$. Then, Eq. (1) can be interpreted as that the second-order origin moments of two generalized incompatible or generalized anti-incompatible operators cannot be arbitrarily small at the same time.

In fact, the remainder $\langle {{\mathscr{C}}}^{\dagger }{\mathscr{C}}\rangle \langle { {\mathcal B} }^{\dagger } {\mathcal B} \rangle$ in the equality (1) reflects the influence of a specific other operator ${\mathscr{C}}$ on the uncertainty relation for ${\mathscr{A}}$ and ${\mathcal B}$. Taking this influence into consideration can make uncertainty relation be expressed as a equality, which is exact to describe the uncertainty relation. To demonstrate the importance of the remainder, an example has been provided in Appendix A to illustrate that the lower bound will be trivial in some special cases if we do not consider the remainder. Most importantly, the remainder plays an important role in the construction of the unified and exact framework, and the corresponding discussion is presented in the next section.

## Unified and Exact Framework for Uncertainty Relations

The unified and exact framework is constructed in this section. In addition to provide a unified description for uncertainty relations, the unified framework can also be used to construct new and stronger uncertainty relations so as to fix deficiencies of previous uncertainty relations and make the uncertainty relation be expressed more accurately.

### Previous uncertainty relations

The Schrödinger uncertainty relation (SUR) is the initial as well as the most widely used product form uncertainty relation^3^:

3 $$\Delta {A}^{2}\Delta {B}^{2}\ge \frac{1}{4}|\langle [A,B]\rangle {|}^{2}+\frac{1}{4}|\langle \{\check{A},\check{B}\}\rangle {|}^{2},$$

where 〈*Q*〉 represents the expected value of *Q*, $\check{Q}=Q-\langle Q\rangle$, and Δ*Q*^2^ = 〈*Q*^2^〉 − 〈*Q*〉^2^ stands for the variance of the observable *Q*. [*A*, *B*] = *AB* − *BA* and $\{\check{A},\check{B}\}=\check{A}\check{B}+\check{B}\check{A}$ represent the commutator and anti-commutator, respectively. One of the most famous sum form uncertainty relations, which have fixed the triviality problem of SUR, takes the form^44^:

4 $$\Delta {A}^{2}+\Delta {B}^{2}\ge |\langle \psi |A\pm iB|{\psi }^{\perp }\rangle {|}^{2}\pm i\langle [A,B]\rangle ,$$

where |*ψ*^⊥^〉 is the state orthogonal to the state of the system |*ψ*〉.

The triviality problem of SUR occurs when the state of the system happens to be the eigenstate of *A* or *B*^39,44^. For instance, one has $|\langle [A,B]\rangle /2{\rm{i}}{|}^{2}+|\langle \{\check{A},\check{B}\}\rangle /2{|}^{2}\equiv \Delta {A}^{2}\Delta {B}^{2}\equiv 0$ in the finite-dimension Hilbert space when Δ*A*^2^ = 0 or Δ*B*^2^ = 0. Different from Δ*A*^2^Δ*B*^2^, the sum of the variances Δ*A*^2^ + Δ*B*^2^ will never be equal to zero for incompatible observables even when the state of the system is an eigenstate of *A* or *B*. Thus, the sum form has the mathematical advantage in expressing the uncertainty relation. However, the lower bounds of the most sum form uncertainty relations rely on the state |*ψ*^⊥^〉, making them difficult to apply to a high dimension Hilbert space^39^. That is to say, previous uncertainty relations in both sum form and product form have deficiencies.

### Uncertainty relation class-C0

The triviality problem can be fixed by the sum form uncertainty relation, but not all sum form uncertainty relations can be used to fix triviality problem. For instance, the product form SUR can also be reformed as a sum form uncertainty relation:

5 $$\Delta {A}^{2}+\Delta {B}^{2}\ge \sqrt{|\langle [A,B]\rangle {|}^{2}+|\langle \{\check{A},\check{B}\}\rangle {|}^{2}},$$

where the inequality Δ*A*^2^ + Δ*B*^2^ ≥ 2Δ*A*Δ*B* has been used. The lower bound (5) turns into zero when Δ*A* = 0 or Δ*B* = 0, and the sum form uncertainty relation (5) therefore has triviality problem. That is to say, the triviality problem cannot be fixed even when SUR is reformed as the sum form. Thus, in addition to the difference in mathematical form, there exists other more essential difference between the product form SUR and the sum form uncertainty relation (4). It is due to this essential difference that the stronger uncertainty relation (4) can fix the triviality problem of SUR. To investigate this difference more clearly, we firstly study SUR.

SUR was initially derived from the Cauchy-Schwarz inequality, and can only be used to describe the uncertainty relation for two incompatible obervables. Since then, lots of work has been done along the way of Schrödinger regime^51–55^, and most of them mainly focused on extending SUR to uncertainty relations for more incompatible observables^56^. We refer to these uncertainty relations as the Schrödinger’s spirit, and these uncertainty relations can be uniformly derived as follows. Assume ${ {\mathcal F} }_{1}=\mathop{\sum }\limits_{m=1}^{N}{x}_{m}{\check{A}}_{m}$, where *A*_*m*_ stands for an arbitrary observable, *N* is the number of the observables and *x*_*m*_ ∈ C represents a random complex number (Without loss of generality, we generally took ${ {\mathcal F} }_{1}=\mathop{\sum }\limits_{m=1}^{N}{e}^{i{\theta }_{m}}{\check{A}}_{m}$). Using the non-negativity of the second-order origin moment of ${ {\mathcal F} }_{1}$^56^:

6 $$\langle { {\mathcal F} }_{1}^{\dagger }{ {\mathcal F} }_{1}\rangle \ge 0,$$

one can obtain:

7 $${\mathbb{D}}\,:\ge 0,$$

where ${\mathbb{D}}$ is a *N* × *N* dimension matrix with the elements ${\mathbb{D}}(m,n)=\langle {\check{A}}_{m}^{\dagger }{\check{A}}_{n}\rangle$ and ${\mathbb{D}}$ : ≥ 0 means that ${\mathbb{D}}$ is a positive semidefinite matrix. As for the positive semidefinite matrix ${\mathbb{D}}$, we have (i) Det(${\mathbb{D}}$) ≥ 0 with Det(${\mathbb{D}}$) being the determinant value of ${\mathbb{D}}$, and (ii) ${X}^{\dagger }.{\mathbb{D}}.X\ge 0$ with *X* ∈ *C*^*N*^ being a random column vector. In fact, utilizing the positive semidefinite matrix ${\mathbb{D}}$, one can construct the uncertainty relations in both product form and sum form, i.e., Det(${\mathbb{D}}$) ≥ 0 turns into the product form uncertainty relation and ${X}^{\dagger }.{\mathbb{D}}.X\ge 0$ becomes the sum form uncertainty relation. For instance, taking *N* = 2 and *X* = {1,*e*^*iθ*^}^*T*^ with 0 ≤ *θ* ≤ 2*π*, one can obtain that (i) Det(${\mathbb{D}}$) ≥ 0 is the SUR and (ii) ${X}^{\dagger }.{\mathbb{D}}.X\ge 0$ is the sum form uncertainty relation (5).

Using the method described above, one can deduce lots of uncertainty relations in both product form and sum form for two and more incompatible observables, and we denote the set of these uncertainty relations as the Class-C0. These two forms uncertainty relations in Class-C0 can both be interpreted as the fundamental inequality $\langle { {\mathcal F} }_{1}^{\dagger }{ {\mathcal F} }_{1}\rangle \ge 0$, and all of them have the triviality problem in expressing the uncertainty relation (for more detail, please see Appendix B). The essential reason for this phenomenon is that the quantum properties of the operator ${ {\mathcal F} }_{1}$, in most cases, cannot be fully expressed by $\langle { {\mathcal F} }_{1}^{\dagger }{ {\mathcal F} }_{1}\rangle \ge 0$, because the non-negativity of the second-order origin moment $\langle { {\mathcal F} }_{1}^{\dagger }{ {\mathcal F} }_{1}\rangle \ge 0$ cannot provide any information of ${ {\mathcal F} }_{1}$ in the quantum level. Thus, to fix the triviality problem, a stronger uncertainty relation class should be constructed.

### Uncertainty relation class-C1

In this subsection, we introduce the concept of “auxiliary operator”. Utilizing this new concept, we construct a stronger uncertainty relation class, which can fix the triviality problem of the Class-C0.

Considering an arbitrary operator ${{\mathscr{O}}}_{1}$, based on Eq. (1), one has:

8 $$\langle { {\mathcal F} }_{1}^{\dagger }{ {\mathcal F} }_{1}\rangle ={ {\mathcal L} }_{1}+\langle { {\mathcal F} }_{2}^{\dagger }{ {\mathcal F} }_{2}\rangle \ge { {\mathcal L} }_{1},$$

where ${ {\mathcal L} }_{1}=(|\langle {[{ {\mathcal F} }_{1},{{\mathscr{O}}}_{1}]}_{{\mathscr{G}}}\rangle {|}^{2}+|\langle {\{{ {\mathcal F} }_{1},{{\mathscr{O}}}_{1}\}}_{{\mathscr{G}}}{\rangle |}^{2})/4|\langle {{\mathscr{O}}}_{1}^{\dagger }{{\mathscr{O}}}_{1}\rangle |$ and ${ {\mathcal F} }_{2}={ {\mathcal F} }_{1}-\langle {{\mathscr{O}}}_{1}^{\dagger }{ {\mathcal F} }_{1}\rangle {{\mathscr{O}}}_{1}/|\langle {{\mathscr{O}}}_{1}^{\dagger }{{\mathscr{O}}}_{1}\rangle |$. In particular, we have ${ {\mathcal L} }_{1} > 0$ when the operator ${{\mathscr{O}}}_{1}$ is generalized incompatible or generalized anti-incompatible with ${ {\mathcal F} }_{1}$. Obviously, the introduction of ${{\mathscr{O}}}_{1}$ provides a more accurate description for the second-order origin moment $\langle { {\mathcal F} }_{1}^{\dagger }{ {\mathcal F} }_{1}\rangle$ that $\langle { {\mathcal F} }_{1}^{\dagger }{ {\mathcal F} }_{1}\rangle \ge 0$ cannot do, and thus we name the operator ${{\mathscr{O}}}_{1}$ as auxiliary operator. In order to investigate the quantum uncertainty relation more accurately, the auxiliary operator should be introduced.

Using (8), we have ${\mathbb{D}}$: ≥ ${{\mathbb{V}}}_{1}$ where ${{\mathbb{V}}}_{1}$ is a *N* × *N* dimension positive semidefinite matrix with the elements ${{\mathbb{V}}}_{1}(m,n)=\langle {\check{A}}_{m}^{\dagger }{{\mathscr{O}}}_{1}\rangle \langle {{\mathscr{O}}}_{1}^{\dagger }{\check{A}}_{n}\rangle /|\langle {{\mathscr{O}}}_{1}^{\dagger }{{\mathscr{O}}}_{1}\rangle |$ and ${\mathbb{D}}$: ≥ ${{\mathbb{V}}}_{1}$ means ${\mathbb{D}}$ − ${{\mathbb{V}}}_{1}$ is a positive semidefinite matrix. Based on the properties of the positive semidefinite matrix ${\mathbb{D}}$ − ${{\mathbb{V}}}_{1}$, we can obtain a series of uncertainty relations for *N* observables in both product form and sum form. For instance, taking *N* = 2, ${{\mathscr{O}}}_{1}=|{\psi }^{\perp }\rangle \langle \psi |$ and *X* = {1, ∓ *i*}^*T*^, one has (i) ${X}^{\dagger }.({\mathbb{D}}-{{\mathbb{V}}}_{1}).X\ge 0$ reduces to the sum form uncertainty relation (4) and (ii) Det(${\mathbb{D}}$ − ${{\mathbb{V}}}_{1}$) ≥ 0 is the product form uncertainty relation $\Delta {{\mathscr{A}}}_{1}^{2}\Delta {{\mathscr{A}}}_{2}^{2}\ge |\langle {[{{\mathscr{A}}}_{1},{{\mathscr{A}}}_{2}]}_{{\mathscr{G}}}\rangle /2i{|}^{2}+|\langle {\{{{\mathscr{A}}}_{1},{{\mathscr{A}}}_{2}\}}_{{\mathscr{G}}}\rangle /2{|}^{2}$ with ${{\mathscr{A}}}_{1}=\check{A}-\langle {\psi }^{\perp }|A|\psi \rangle |{\psi }^{\perp }\rangle \langle \psi |$ and ${{\mathscr{A}}}_{2}=\check{B}-\langle {\psi }^{\perp }|B|\psi \rangle |{\psi }^{\perp }\rangle \langle \psi |$.

Similar to Class-C0, we denote the set of the uncertainty relations, that can be directly deduced from (8), as the Class-C1. In fact, the Class-C1 is the set of uncertainty relations which can be obtained by introducing one auxiliary operator and the Class-C0 is the set of the uncertainty relations without considering the auxiliary operator. Due to the existence of auxiliary operator, the Class-C1 can provide more accurate description for uncertainty relation, and thus the Class-C1 is stronger than the Class-C0. Based on the discussion above, the uncertainty relation (4) can be considered as taking the non-Hermitian operator |*ψ*^⊥^〉〈*ψ*| as the auxiliary operator, and the uncertainty relation (4) therefore belongs to the stronger Class-C1.

We then show that the triviality problem of Class-C0 can be fixed by the stronger Class-C1. The triviality problem of Class-C0 occurs when the state of the system happens to be the eigenstate of one of the incompatible observables^39,44^. For instance, as for SUR in Class-C0, the triviality problem occurs when Δ*A*^2^ = 0 or Δ*B*^2^ = 0. In fact, the physical essence of the triviality problem can be described as that we cannot obtain any information of the uncertainty of A(B), when the state of the system happens to be an eigenstate of B(A). Thus, the auxiliary operator, which can provide a more accurate description for the uncertainty relation, can be used to fix this triviality problem. According to (1) and (8), the auxiliary operator ${{\mathscr{O}}}_{1}$ will not provide any effective description for ${ {\mathcal F} }_{1}$ when $\langle {{\mathscr{O}}}_{1}^{\dagger }{{\mathscr{O}}}_{1}\rangle =0$, and thus the auxiliary operator introduced to fix the triviality problem should satisfy $\langle {{\mathscr{O}}}_{1}^{\dagger }{{\mathscr{O}}}_{1}\rangle \ne 0$ (for more detail, please see Appendix C). In fact, this deduction can be used to explain that why the triviality problem is fixed by the uncertainty relation (4). The uncertainty relation (4) can be considered as taking |*ψ*^⊥^〉〈*ψ*| as the auxiliary operator. The second-order origin moment of |*ψ*^⊥^〉〈*ψ*| is never equal to zero, and the triviality problem can therefore be fixed by the uncertainty relation (4). In addition to uncertainty relation (4), the other sum form uncertainty relations in Class-C1, which are obtained by introducing an auxiliary operator with non-zero second-order origin moment, can also fix the triviality problem.

However, in Class-C1, the uncertainty relations that can be used to fix the triviality problem of the Class-C0 generally have other deficiency, and this deficiency is actually the inevitable result for fixing the triviality problem. In order to fix the triviality problem, the auxiliary operator usually should be state-dependent so as to guarantee its second-order origin moment be not equal to zero for all quantum states. However, such a state-dependent auxiliary operator is usually difficult to be obtained for some special quantum states, which leads to the uncertainty relation cannot be well applied to these quantum states. For instance, the uncertainty relation (4) fix the triviality problem by taking the state-dependent operator |*ψ*^⊥^〉〈*ψ*| as the auxiliary operator. However, it is due to the existence of |*ψ*^⊥^〉〈*ψ*| that the uncertainty relation (4) cannot be well applied to a high dimension system. Thus, both the Class-C0 and the stronger Class-C1 have deficiencies in expressing uncertainty relation.

### Uncertainty relation class-C2

The uncertainty relation Class-C0 and Class-C1 are the set of the uncertainty relations with zero and one auxiliary operator, respectively. The two classes can recover some well-known previous uncertainty relations, but both of them have deficiencies. Then, we wonder that: can we introduce more auxiliary operators so as to construct a stronger uncertainty relation class, and if so, can the deficiencies of the Class-C0 and Class-C1 be completely fixed by the new stronger class? We denote the uncertainty relations class with *m* auxiliary operators by Class-Cm with *m* being arbitrary positive integer. In this subsection, we firstly introduce the Class-C2, and then show that the deficiencies of the Class-C0 and Class-C1 can be completely fixed by the stronger Class-C2.

For the construction of the Class-C1, the remainder $\langle { {\mathcal F} }_{2}^{\dagger }{ {\mathcal F} }_{2}\rangle$ in (8) has been ignored. To construct stronger uncertainty relation class, this remainder $\langle { {\mathcal F} }_{2}^{\dagger }{ {\mathcal F} }_{2}\rangle$ should be considered. Assuming an arbitrary operator ${{\mathscr{O}}}_{2}$ and taking advantage of Eq. (1), one can obtain:

9 $$\langle { {\mathcal F} }_{2}^{\dagger }{ {\mathcal F} }_{2}\rangle ={ {\mathcal L} }_{2}+\langle { {\mathcal F} }_{3}^{\dagger }{ {\mathcal F} }_{3}\rangle ,$$

where ${ {\mathcal L} }_{2}=(|\langle {[{ {\mathcal F} }_{2},{{\mathscr{O}}}_{2}]}_{{\mathscr{G}}}\rangle {|}^{2}+|\langle {\{{ {\mathcal F} }_{2},{{\mathscr{O}}}_{2}\}}_{{\mathscr{G}}}{\rangle |}^{2})/4|\langle {{\mathscr{O}}}_{2}^{\dagger }{{\mathscr{O}}}_{2}\rangle |$ and ${ {\mathcal F} }_{3}={ {\mathcal F} }_{2}-\langle {{\mathscr{O}}}_{2}^{\dagger }{ {\mathcal F} }_{2}\rangle {{\mathscr{O}}}_{2}/|\langle {{\mathscr{O}}}_{2}^{\dagger }{{\mathscr{O}}}_{2}\rangle |$. Taking (9) into (8), one can introduce another auxiliary operator:

10 $$\langle { {\mathcal F} }_{1}^{\dagger }{ {\mathcal F} }_{1}\rangle ={ {\mathcal L} }_{1}+{ {\mathcal L} }_{2}+\langle { {\mathcal F} }_{3}^{\dagger }{ {\mathcal F} }_{3}\rangle \ge { {\mathcal L} }_{1}+{ {\mathcal L} }_{2},$$

using (10), we can construct lots of uncertainty relations in both product form and sum form (The sum form uncertainty relations can be obtained by directly taking ${ {\mathcal F} }_{1}=\mathop{\sum }\limits_{m=1}^{N}{x}_{m}{\check{A}}_{m}$ into Eq. (10). To construct the product form uncertainty relations, the auxiliary operators involved should satisfy the conditions that $\langle {{\mathscr{O}}}_{k}^{\dagger }{{\mathscr{O}}}_{l}\rangle =\langle {{\mathscr{O}}}_{k}^{\dagger }{{\mathscr{O}}}_{l}\rangle {\delta }_{kl}$ and $\langle {{\mathscr{O}}}_{k}^{\dagger }{{\mathscr{O}}}_{k}\rangle \ne 0$. Equation (10) will be reformed as ${\mathbb{D}}:\ge \mathop{\sum }\limits_{k=1}^{2}{{\mathbb{V}}}_{k}$ when the conditions above are satisfied (see Appendix D). Taking advantage of ${\mathbb{D}}\ge \mathop{\sum }\limits_{k=1}^{2}{{\mathbb{V}}}_{k}$, one can construct the product form uncertainty relations. The similar deduction can be easily extended to the case for *m* ≥ 2, namely Eq. (12). Remarkably, the auxiliary operators does not need to satisfy conditions above when the uncertainty relations in the Class-Cm are not required to writen as product form), and these uncertainty relations, which involve two auxiliary operators, constitute the Class-C2.

**Figure 2:**
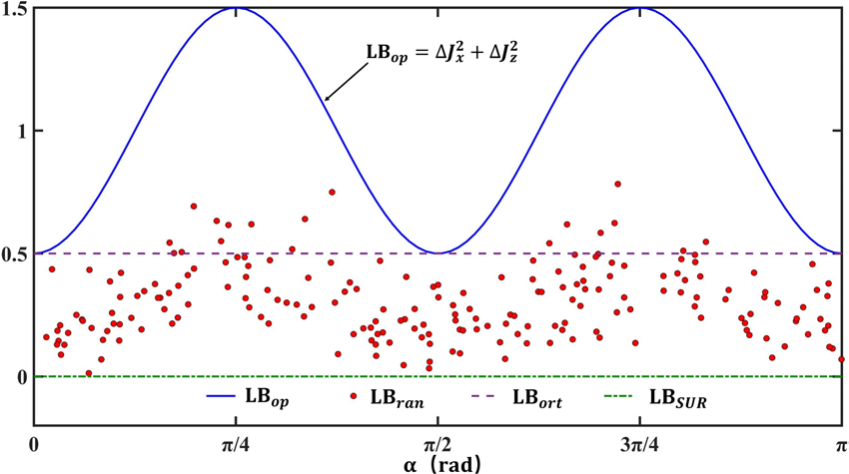
The spin-1 system is chosen as the platform to demonstrate the new uncertainty inequality (11). We take *A* = *J*_*x*_, *B* = *J*_*z*_, $\hslash$ = 1, and the state is parameterized by *α* as *ρ* = cos^2^(*α*)|1〉〈1| + sin^2^(*α*)|−1〉〈−1|, with |±1〉 and |0〉 being the eigenstates of *J*_*z*_. The green dash-dotted line represents the lower bound of the SUR (denoted by *LB*_*SUR*_). It can be seen that the lower bound of the SUR is trivial all the time. According to ref. ^44^, the uncertainty relation (4) turns into Δ*A*^2^ + Δ*B*^2^ ≥ ∓ *i*〈[*A*, *B*]〉 + 〈(−*A* ± *iB*|*ψ*^⊥^〉〈*ψ*^⊥^|(−*A* ∓ *iB*)〉 for the mixed state ${\rho }_{mixed}=\sum {p}_{j}|{\psi }_{j}\rangle \langle {\psi }_{j}|$, if there exists a state |*ψ*^⊥^〉 orthogonal to all states |*ψ*_*j*_〉. Obviously, the orthogonal state |*ψ*^⊥^〉 can only be taken as |0〉 for the given state *ρ*, and the corresponding bound is noted by the purple dashed line (denoted by *LB*_*ort*_). The 200 red dots (denoted by *LB*_*ran*_) stand for the lower bound (11) which are calculated by randomly taking 200 sets of *α*, ${\mathcal R}$ and ${\mathscr{S}}$ into (11). The blue solid line is the optimal lower bound of (11) (denoted by *LB*_*op*_), which is obtained by taking ${\mathcal R} ={\lambda }_{1}\check{A}+{\lambda }_{2}\check{B}$ with |*λ*_1_|^2^ = |*λ*_2_|^2^ ≠ 0. We can find that *LB*_*op*_ is exactly equal to the sum of the uncertainties Δ*J*_*x*_^2^ + Δ*J*_*z*_^2^.

As mentioned in the previous subsection, the triviality problem can be fixed when the second-order origin moment of the auxiliary operator is not equal to zero. According to Eq. (1), the second-order origin moments of two operators will never be zero at the same time when they are generalized incompatible or anti-incompatible with each other. Hence, at least one of the two auxiliary operators can be used to fix the triviality problem, when we take the two generalized incompatible operators as the auxiliary operators. For instance, assuming two generalized incompatible operators ${\mathcal R}$ and ${\mathscr{S}}$, and taking ${{\mathscr{O}}}_{1}= {\mathcal R}$, ${{\mathscr{O}}}_{2}={\mathscr{S}}$ and *N* = 2, one can obtain:

11 $$\Delta {A}^{2}+\Delta {B}^{2}\ge { {\mathcal L} }_{ {\mathcal R} }+{ {\mathcal L} }_{{\mathscr{S}}}-\langle {\{\check{A},{e}^{i\theta }\check{B}\}}_{{\mathscr{G}}}\rangle ,$$

where $${ {\mathcal L} }_{ {\mathcal R} }=(|{[(\check{A}+{e}^{i\theta }\check{B}), {\mathcal R} ]}_{{\mathscr{G}}}{|}^{2}+|{\{(\check{A}+{e}^{i\theta }\check{B}), {\mathcal R} \}}_{{\mathscr{G}}}{|}^{2})/(4|\langle { {\mathcal R} }^{\dagger } {\mathcal R} \rangle |),{ {\mathcal L} }_{{\mathscr{S}}}=(|{[{ {\mathcal F} }_{{\mathscr{S}}},{\mathscr{S}}]}_{{\mathscr{G}}}{|}^{2}+|{\{{ {\mathcal F} }_{{\mathscr{S}}},{\mathscr{S}}\}}_{{\mathscr{G}}}{|}^{2})/$$$$(4|\langle {{\mathscr{S}}}^{\dagger }{\mathscr{S}}\rangle |)$$, $${ {\mathcal F} }_{{\mathscr{S}}}=\check{A}+{e}^{i\theta }\check{B}-\langle { {\mathcal R} }^{\dagger }(\check{A}+{e}^{i\theta }\check{B})\rangle  {\mathcal R} /|\langle { {\mathcal R} }^{\dagger } {\mathcal R} \rangle |$$, and *θ* should be chosen to maximize the lower bound. The triviality problem can be completely fixed by the uncertainty relation (11) for almost any choice of the generalized incompatible operators $$ {\mathcal R} $$ and $${\mathscr{S}}$$: choose $$ {\mathcal R} $$ and $${\mathscr{S}}$$ that can avoid $$\langle \check{A}\check{B}\rangle \equiv \langle { {\mathcal R} }^{\dagger }\check{A}\rangle \equiv \langle { {\mathcal R} }^{\dagger }\check{B}\rangle \equiv \langle {{\mathscr{S}}}^{\dagger }\check{A}\rangle \equiv $$$$\langle {{\mathscr{S}}}^{\dagger }\check{B}\rangle \equiv 0$$ (for more detail, please see Appendix C). Such a choice is always possible, as shown in Fig. 2.

Equation (1) indicates that the second-order origin moments of two generalized incompatible operators cannot be arbitrarily small at the same time, which constitutes the basic idea for fixing the triviality problem by introducing two generalized incompatible auxiliary operators. This physical phenomenon revealed by Eq. (1) applies for all quantum states, and does not rely on the state of the system. Thus, the uncertainty relations, which fix the triviality problem by introducing two generalized incompatible operators, can be well applied to arbitrary quantum state. In particular, the uncertainty relation (11) does not rely on |*ψ*^⊥^〉, and it therefore can be well applied to a high dimension system. Moreover, the uncertainty relation (11) has a tighter lower bound than the uncertainty relation depending on |*ψ*^⊥^〉 by limiting the choice of the auxiliary operators, as shown in Fig. 2. Also, the inequality (11) will become an equality on the condition that ${\mathcal R} ={\lambda }_{1}\check{A}+{\lambda }_{2}\check{B}$ with |*λ*_1_|^2^ = |*λ*_2_|^2^ ≠ 0 and *λ*_1_,*λ*_2_ ∈ *C*. The condition is independent on the state |*ψ*^⊥^〉, and can therefore be easily satisfied even for a high dimension Hilbert space. Hence, by introducing two generalized incompatible auxiliary operators, the deficiencies in the Class-C0 and Class-C1 can be completely fixed by the sum form uncertainty relations in the stronger Class-C2.

### Uncertainty relation class-Cm

According to (9) and (10), we can see that the remainder of Eq. (1) plays an important role in the construction of the Class-C2, since it can be used to introduce the auxiliary operator ${{\mathscr{O}}}_{2}$. As shown in (10), a new remainder $\langle { {\mathcal F} }_{3}^{\dagger }{ {\mathcal F} }_{3}\rangle$ appears with the introduction of the auxiliary operator ${{\mathscr{O}}}_{2}$. Similar to (9), using the new appeared remainder, we can introduce another auxiliary operator ${{\mathscr{O}}}_{3}$. By constantly iterating the remainder, one can introduce any number of auxiliary operators we need. Thus, the uncertainty relation Class-Cm is constructed:

12 $$\langle { {\mathcal F} }_{1}^{\dagger }{ {\mathcal F} }_{1}\rangle =\mathop{\sum }\limits_{k=1}^{m}{ {\mathcal L} }_{k}+\langle { {\mathcal F} }_{m+1}^{\dagger }{ {\mathcal F} }_{m+1}\rangle \ge \mathop{\sum }\limits_{k=1}^{m}{ {\mathcal L} }_{k},$$

where ${ {\mathcal L} }_{k}=(|\langle {[{ {\mathcal F} }_{k},{{\mathscr{O}}}_{k}]}_{{\mathscr{G}}}\rangle {|}^{2}+|\langle {\{{ {\mathcal F} }_{k},{{\mathscr{O}}}_{k}\}}_{{\mathscr{G}}}{\rangle |}^{2})/4|\langle {{\mathscr{O}}}_{k}^{\dagger }{{\mathscr{O}}}_{k}\rangle |$ and ${ {\mathcal F} }_{k+1}={ {\mathcal F} }_{k}-\langle {{\mathscr{O}}}_{k}^{\dagger }{ {\mathcal F} }_{k}\rangle {{\mathscr{O}}}_{k}/|\langle {{\mathscr{O}}}_{k}^{\dagger }{{\mathscr{O}}}_{k}\rangle |$, with ${{\mathscr{O}}}_{k}$ being an arbitrary auxiliary operator. Utilizing (12), one can construct lots of uncertainty relations in both product form and sum form for two and more incompatible observables (The sum form uncertainty relations can be obtained by directly taking ${ {\mathcal F} }_{1}=\mathop{\sum }\limits_{m=1}^{N}{x}_{m}{\check{A}}_{m}$ into Eq. (10). To construct the product form uncertainty relations, the auxiliary operators involved should satisfy the conditions that $\langle {{\mathscr{O}}}_{k}^{\dagger }{{\mathscr{O}}}_{l}\rangle =\langle {{\mathscr{O}}}_{k}^{\dagger }{{\mathscr{O}}}_{l}\rangle {\delta }_{kl}$ and $\langle {{\mathscr{O}}}_{k}^{\dagger }{{\mathscr{O}}}_{k}\rangle \ne 0$. Equation (10) will be reformed as ${\mathbb{D}}:\ge \mathop{\sum }\limits_{k=1}^{2}{{\mathbb{V}}}_{k}$ when the conditions above are satisfied (see Appendix D). Taking advantage of ${\mathbb{D}}\ge \mathop{\sum }\limits_{k=1}^{2}{{\mathbb{V}}}_{k}$, one can construct the product form uncertainty relations. The similar deduction can be easily extended to the case for *m* ≥ 2, namely Eq. (12). Remarkably, the auxiliary operators does not need to satisfy conditions above when the uncertainty relations in the Class-Cm are not required to writen as product form.), and these uncertainty relations constitute the uncertainty relation Class-Cm. Also, the deficiencies in the Class-C0 and Class-C1 can be completely fixed by the sum form uncertainty relations in the Class-Cm when there exist two generalized incompatible or generalized anti-incompatible operators in the *m* auxiliary operators.

**Figure 3:**
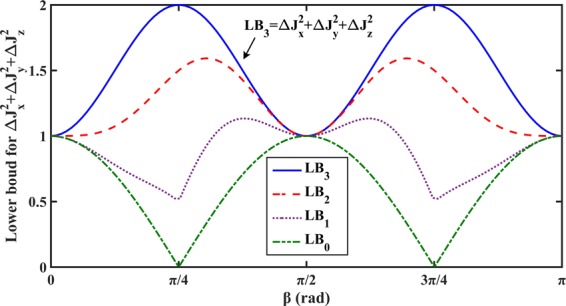
Illustration to demonstrate the function of the auxiliary operator is presented. We take $\hslash$ = 1, and assume that the state of the spin-1 system is in the pure state |*ψ*〉 = cos(*β*)|1〉 + sin(*β*)|−1〉 with |±1〉 being the eigenstates of *J*_*z*_. By introducing different number of auxiliary operators, we can obtain a series of sum form uncertainty relations: $\Delta {J}_{x}^{2}+\Delta {J}_{y}^{2}+\Delta {J}_{z}^{2}\ge L{B}_{m}=\mathop{\sum }\limits_{k=1}^{m}\,{ {\mathcal L} }_{k}-\langle {\{{e}^{i{\theta }_{1}}{J}_{x},{e}^{i{\theta }_{2}}{J}_{y}\}}_{{\mathscr{G}}}\rangle -\langle {\{{e}^{i{\theta }_{2}}{J}_{y},{e}^{i{\theta }_{3}}{J}_{z}\}}_{{\mathscr{G}}}\rangle -\langle {\{{e}^{i{\theta }_{3}}{J}_{z},{e}^{i{\theta }_{1}}{J}_{x}\}}_{{\mathscr{G}}}\rangle$ with ${ {\mathcal L} }_{k}=(|\langle {[({e}^{i{\theta }_{1}}{J}_{x}+{e}^{i{\theta }_{2}}{J}_{y}+{e}^{i{\theta }_{3}}{J}_{z}),{{\mathscr{O}}}_{k}]}_{{\mathscr{G}}}\rangle {|}^{2}+|\langle {\{({e}^{i{\theta }_{1}}{J}_{x}+{e}^{i{\theta }_{2}}{J}_{y}+{e}^{i{\theta }_{3}}{J}_{z}),{{\mathscr{O}}}_{k}\}}_{{\mathscr{G}}}\rangle {|}^{2})/4|\langle {{\mathscr{O}}}_{k}^{\dagger }{{\mathscr{O}}}_{k}\rangle |$. The auxiliary operator ${{\mathscr{O}}}_{k}$ belongs to the set $\Theta =\{{{\mathscr{O}}}_{1},{{\mathscr{O}}}_{2},{{\mathscr{O}}}_{3}\}$, which is obtained by the Schmidt transformation (see the Schmidt transformation process in Appendix E). We can see that the tightness of the uncertainty relation becomes better and better with *m* increasing, and the uncertainty inequality will become an equality when *m* = 3. The uncertainty relation becomes $\Delta {J}_{x}^{2}+\Delta {J}_{y}^{2}+\Delta {J}_{z}^{2}\ge L{B}_{0}=-\langle {\{{e}^{i{\theta }_{1}}{J}_{x},{e}^{i{\theta }_{2}}{J}_{y}\}}_{{\mathscr{G}}}\rangle -\langle {\{{e}^{i{\theta }_{2}}{J}_{y},{e}^{i{\theta }_{3}}{J}_{z}\}}_{{\mathscr{G}}}\rangle -\langle {\{{e}^{i{\theta }_{3}}{J}_{z},{e}^{i{\theta }_{1}}{J}_{x}\}}_{{\mathscr{G}}}\rangle$ when we do not take the auxiliary operators into consideration. It is clear that the tightness of *LB*_0_ is worse than the other uncertainty relations, and *LB*_0_ is trivial when *β* = *π*/4 and 3*π*/4.

As we known, tightness is an important index to measure the quality of the uncertainty relation^12,40,45,57,58^, because the uncertainty relation with tighter lower bound generally has wider application. Thus, lots of work has been done to improve the tightness of the uncertainty relation^39^. The tighter lower bound essentially means that the lower bound can provide more accurate description for the uncertainty relation. The function of the auxiliary operator is to provide more accurate description for the uncertainty relation, and the lower bound therefore becomes tighter and tighter with the introduction of auxiliary operators, as shown in Fig. 3. Thus, we can construct uncertainty relation with any tightness we need by controlling the number of the auxiliary operators introduced.

### Exact uncertainty relation class-Cr

The uncertainty relation is essentially to investigate the relationship between the uncertainties of incompatible observables, and this relationship is in general expressed by the inequality, which is not exact. As mentioned above, the uncertainty relation can be expressed more accurately with the introduction of the auxiliary operators. We then show that the uncertainty relation can be exactly expressed by an equality when *r* auxiliary operators, which satisfy a given condition, are introduced, as shown in Fig. 3. The value of *r* is equal to the rank of the Metric matrix corresponding to the bilinear operator function ${{\mathscr{A}}}^{+} {\mathcal B}$ (for more detail, please see Appendix D). That is to say, we can construct a Class-Cr, which is exact in descripting uncertainty relation.

Taking advantage of Eq. (1), one can obtain (for more detail, please see Appendix D):

13 $$\langle { {\mathcal F} }_{1}^{\dagger }{ {\mathcal F} }_{1}\rangle =\mathop{\sum }\limits_{k=1}^{m}{ {\mathcal L} }_{k}$$

where ${{\mathscr{O}}}_{k}$ is the element of the operator set $\Theta =\{{{\mathscr{O}}}_{1},{{\mathscr{O}}}_{2},\ldots ,{{\mathscr{O}}}_{r}\}$ in which the elements satisfy $\langle {{\mathscr{O}}}_{i}^{\dagger }{{\mathscr{O}}}_{j}\rangle =\langle {{\mathscr{O}}}_{i}^{\dagger }{{\mathscr{O}}}_{j}\rangle {\delta }_{ij}$ and $\langle {{\mathscr{O}}}_{k}^{\dagger }{{\mathscr{O}}}_{k}\rangle \ne 0$ with *k*, *i*, *j* ∈ {1,2,…,*r*}. The set can be obtained by the Schmidt transformation (please see Appendix E). In general, ${ {\mathcal L} }_{k}=(|\langle {[{ {\mathcal F} }_{k},{{\mathscr{O}}}_{k}]}_{{\mathscr{G}}}\rangle {|}^{2}+|\langle {\{{ {\mathcal F} }_{k},{{\mathscr{O}}}_{k}\}}_{{\mathscr{G}}}{\rangle |}^{2})/4|\langle {{\mathscr{O}}}_{k}^{\dagger }{{\mathscr{O}}}_{k}\rangle |$. Here, ${ {\mathcal L} }_{k}$ can be simplified as $(|\langle {[{ {\mathcal F} }_{1},{{\mathscr{O}}}_{k}]}_{{\mathscr{G}}}\rangle {|}^{2}+|\langle {\{{ {\mathcal F} }_{1},{{\mathscr{O}}}_{k}\}}_{{\mathscr{G}}}\rangle {|}^{2})/4|\langle {{\mathscr{O}}}_{k}^{\dagger }{{\mathscr{O}}}_{k}\rangle |$ when the auxiliary operators satisfy the conditions mentioned above. Indeed, the value of *r* is the maximum number of the operators one can find, which satisfy $\langle {{\mathscr{O}}}_{i}^{\dagger }{{\mathscr{O}}}_{j}\rangle =\langle {{\mathscr{O}}}_{i}^{\dagger }{{\mathscr{O}}}_{j}\rangle {\delta }_{ij}$ and $\langle {{\mathscr{O}}}_{k}^{\dagger }{{\mathscr{O}}}_{k}\rangle \ne 0$. *r* only depends on the state of the system. It is worth mentioning that *r* is less than *d* for the pure state and less than *d*^2^ for the mixed state in the *d*-dimension system, and *r* will tend to the infinity when considering the infinite-dimension system^59–64^.

In mathematics, the tighter uncertainty relation essentially means that the lower bound of the uncertainty relation is “closer” to the left-hand side of the uncertainty relation. The bound of the uncertainty equality is exactly equal to the left-hand side of the uncertainty relation, which means the bound of the uncertainty equality is “closest” to the left-hand side of the uncertainty relation. Thus, the uncertainty equality can be considered as providing the tightest lower bound for the uncertainty relation. In physics, the tighter uncertainty relation means the lower bound can provide more accurate description for the uncertainty relation. Thus, we say that the tightest lower bound provided by the uncertainty equality, is exact in describing the uncertainty relation. In the next section, we will show that, due to possessing the tightest lower bound, the uncertainty equality has more effective application than the uncertainty inequality.

Remarkably, the discussion above indicates that the uncertainty relation can be exactly described by an equality when *r* auxiliary operators satisfying a given condition are introduced, but it does not mean that at most *r* auxiliary operators can be introduced. We can introduce any number of auxiliary operators we want when the auxiliary operators are not required to meet the given conditions.

## Application in Non-Markovianity Witness

Uncertainty relation has been widely used in quantum information science, and most of these applications require tighter lower bounds. The uncertainty relation becomes tighter and tighter with the introduction of auxiliary operators, and thus the uncertainty relations with more auxiliary operators, namely the uncertainty relations in the stronger uncertainty relation classes, have more effective application.

In this section, taking the dephasing channel as the illustration, we show that the uncertainty relation with more auxiliary operators can be used to witness the non-Markovian dynamics more effectively. Reference ^65^ showed that dynamics of a system is non-Markovian when the uncertainty relation for the Choi state corresponding to this system is violated. The uncertainty relation with tighter lower bound is violated more easily than the regular uncertainty relation, because the tighter lower bound is “closer” to the left-hand side of the uncertainty relation. Thus, the uncertainty relation with more auxiliary operators, which possesses tighter lower bound, is more effective to witness the non-Markovian dynamics.

The Lindblad equation for a pure dephasing channel is given by^65,66^:

14 $$\frac{d}{dt}{\rho }_{t}=\gamma (t)({\sigma }_{z}{\rho }_{t}{\sigma }_{z}-{\rho }_{t}),$$

where $$\gamma (t)$$ reads:

15 $$\gamma (t)=\frac{2\lambda {\gamma }_{0}\,\sinh (tg/2)}{g\,\cosh (tg/2)+\lambda \,\sinh (tg/2)},$$

with $g=\sqrt{{\lambda }^{2}-2{\gamma }_{0}\lambda }$. *λ* and *γ*_0_ are two positive constants related to the reservoir^66^. *γ*(*t*) is the time-dependent dephasing rate determined by the spectral density of the reservoir^67^, and the dynamics of the system is non-Markovian when *γ*(*t*) < 0^65^.

**Figure 4:**
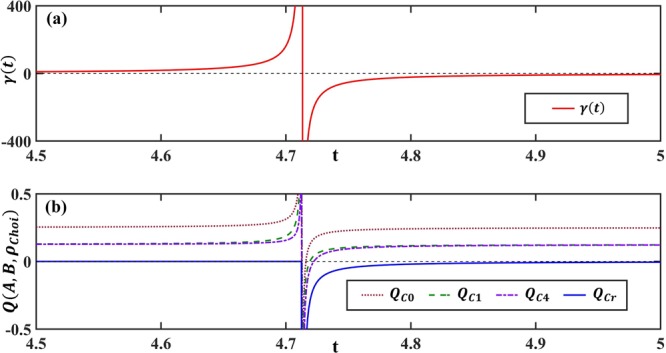
Evolutions of *γ*(*t*) and *Q*(*A*,*B*,*ρ*_*Choi*_) with respect to time *t* are demonstrated in (**a**) and (**b**), respectively (*A* is taken as $$(\begin{array}{llll}1 & 0 & 0 & 0\\ 0 & 0 & 0 & 0\\ 0 & 0 & 0 & 0\\ 0 & 0 & 0 & 0\end{array})$$, and *B* is taken as $$(\begin{array}{llll}0 & 0 & 0 & 1\\ 0 & 0 & 1 & 0\\ 0 & 1 & 0 & 0\\ 1 & 0 & 0 & 0\end{array})$$). We take *λ* = *γ*_0_, *γ*_0_ = 1, and *ε* = 10^−4^. Here, the uncertainty relations, that are used to detect non-Markovianity, are taken as Δ*A*^2^ + Δ*B*^2^ ≥ *i*〈[*A*, *B*]〉, $$\Delta {A}^{2}+\Delta {B}^{2}\ge { {\mathcal L} }_{Choi}^{(1)}+i\langle [A,B]\rangle $$, $$\Delta {A}^{2}+\Delta {B}^{2}\ge \mathop{\sum }\limits_{k=1}^{4}\,{ {\mathcal L} }_{Choi}^{(k)}+i\langle [A,B]\rangle $$, and $$\Delta {A}^{2}+\Delta {B}^{2}=\mathop{\sum }\limits_{k=1}^{r}\,{ {\mathcal L} }_{Choi}^{(k)}+i\langle [A,B]\rangle $$, with $${ {\mathcal L} }_{Choi}^{(k)}=(|\langle {[({\check{A}}+i{\check{B}}),{{\mathscr{O}}}_{Choi}^{k}]}_{{\mathscr{G}}}\rangle {|}^{2}+|\langle {\{({\check{A}}+i{\check{B}}),{{\mathscr{O}}}_{Choi}^{k}\}}_{{\mathscr{G}}}\rangle {|}^{2})$$$$/4|\langle {{\mathscr{O}}}_{Choi}^{k}{}^{\dagger }{{\mathscr{O}}}_{Choi}^{k}\rangle |$$. The auxiliary operator set $$\{{{\mathscr{O}}}_{Choi}^{1},{{\mathscr{O}}}_{Choi}^{2},\cdots ,{{\mathscr{O}}}_{Choi}^{r}\}$$ with *r* = 8 can be obtained by Schmidt transformation. $${Q}_{Cm}=\Delta {A}^{2}+\Delta {B}^{2}-(\mathop{\sum }\limits_{k=1}^{m}\,{ {\mathcal L} }_{Choi}^{(k)}+i\langle [A,B]\rangle )$$ is the *Q*(*A*,*B*,*ρ*_*Choi*_) related to the uncertainty relation $$\Delta {A}^{2}+\Delta {B}^{2}\ge \mathop{\sum }\limits_{k=1}^{m}\,{ {\mathcal L} }_{Choi}^{(k)}+i\langle [A,B]\rangle $$. The uncertainty relation is violated when the corresponding *Q*_*Cm*_ is less than zero. The uncertainty relation $$\Delta {A}^{2}+\Delta {B}^{2}\ge \mathop{\sum }\limits_{k=1}^{m}\,{ {\mathcal L} }_{Choi}^{(k)}+i\langle [A,B]\rangle $$ involves *m* auxiliary operators, and it therefore belongs to the uncertainty relation Class-Cm. We can see that the uncertainty relation involving more auxiliary operators can be used to detect the non-Markovianity more effectively. In particular, in *t* = 4.8, we have *γ*(*t*) < 0 which means the dynamics is non-Markovian, and we can see that the non-Markovianity around this moment can only be witnessed by the uncertainty equality (The uncertainty relation turns into an equality $$\Delta {A}^{2}+\Delta {B}^{2}=-\langle {\{{\check{A}},{e}^{i\theta }{\check{B}}\}}_{{\mathscr{G}}}\rangle $$ when the second-order origin moment of $${\mathscr{A}}$$ is equal to zero. Since Δ*A*^2^ + Δ*B*^2^ is never equal to zero when there does not exist common eigenstate between *A* and *B*, the lower bound of uncertainty equality is guranteed to be non-trivial).

In order to witness the non-Markovianity by the uncertainty relation, we define the following quantity:

16 $$Q(A,B,{\rho }_{Choi})=\Delta {A}^{2}+\Delta {B}^{2}-L{B}_{s},$$

where *ρ*_*Choi*_ is the Choi state corresponding to the system, and *LB*_*s*_ represents the lower bound of the sum form uncertainty relation Δ*A*^2^ + Δ*B*^2^ ≥ *LB*_*s*_. Here we should mention that the uncertainty relation is calculated on the Choi state *ρ*_*Choi*_, and the introduction of the Choi state is presented in Appendix F. According to the discussion above, we have that the dynmics is non-Markovian when *Q*(*A*,*B*,*ρ*_*Choi*_) < 0^65^. The evolutions of *γ*(*t*) and *Q*(*A*,*B*,*ρ*_*Choi*_) are presented in Fig. 4. We can see that the non-Markovianity can be witnessed more effectively with the introduction of auxiliary operators.

In particular, the uncertainty inequality becomes the uncertainty equality when *r* auxiliary operators are introduced. As mentioned above, the uncertainty equality possesses the tightest lower bound, and thus the uncertainty equality is more effective than the uncertainty inequality in the non-Markovianity witness, as shown in Fig. 4.

## The Applicability of the Unified and Exact Framework to Non-Hermitian Operators

There exist two kinds of operators in quantum mechanics: Hermitian and non-Hermitian operators, but it should be paid particular attention that the previous uncertainty relations are contradictory with the non-Hermitian operators, i.e., lots of uncertainty relations will be violated when applied to non-Hermitian operators^68–70^. For instance, we have $|[{\sigma }_{+},{\sigma }_{-}]{|}^{2}/4+|\{{\sigma }_{+},{\sigma }_{-}\}{|}^{2}/4\ge \Delta {{\sigma }_{+}}^{2}\Delta {{\sigma }_{-}}^{2}$ for all qubit systems, where the non-Hermitian operator *σ* + (*σ*_−_) is the raising (lowering) operator of the single qubit system. That is to say, different from the Hermitian operators, the uncertainties of the non-Hermitian operators are not lower-bounded by the quantities related with the commutator and anti-commutator. The essential reason for this phenomenon is that $i[{\mathscr{A}}, {\mathcal B} ]$ and $\{{\mathscr{A}}, {\mathcal B} \}$ cannot be guaranteed to be Hermitian by the existing definition of commutator and anti-commutator when the operator ${\mathscr{A}}$ or ${\mathcal B}$ is non-Hermitian. The new definition of the generalized commutator and generalized anti-commutator can guarantee that the $i{[{\mathscr{A}}, {\mathcal B} ]}_{{\mathscr{G}}}$ and ${\{{\mathscr{A}}, {\mathcal B} \}}_{{\mathscr{G}}}$ are Hermitian for both Hermitian and non-Hermitian operators, and thus the contradiction between the uncertainty relations and the non-Hermitian operators is fixed.

As mentioned above, Eq. (1) indicates that the second-order origin moments $\langle {{\mathscr{A}}}^{\dagger }{\mathscr{A}}\rangle$ and $\langle { {\mathcal B} }^{\dagger } {\mathcal B} \rangle$ cannot be arbitrarily small at the same time when ${\mathscr{A}}$ and ${\mathcal B}$ are generalized incompatible or generalized anti-incompatible with each other. The interpretation of (1) reveals some novel quantum properties that the previous uncertainty relations cannot do. Such as, applying the Eq. (1) to the annihilation operators *a*_1_ and *a*_2_ of two continuous variable subsystems, one can deduce that the product of the expected energy of two subsystems $\langle {a}_{1}^{\dagger }{a}_{1}\rangle \langle {a}_{2}^{\dagger }{a}_{2}\rangle$ is lower-bounded by $|\langle {[{a}_{1},{a}_{2}]}_{{\mathscr{G}}}\rangle {|}^{2}/4+|\langle {\{{a}_{1},{a}_{2}\}}_{{\mathscr{G}}}\rangle {|}^{2}/4$. In particular, the energy of two subsystems cannot be arbitrarily small at the same time, when the annihilation operators of the two systems are generalized incompatible or generalized anti-incompatible on the state of the system, which means $\langle {[{a}_{1},{a}_{2}]}_{{\mathscr{G}}}\rangle$ or $\langle {\{{a}_{1},{a}_{2}\}}_{{\mathscr{G}}}\rangle$ does not equal or tend to zero.

## Discussion

We provide a unified and exact framework for uncertainty relations, and the uncertainty relations in this unified framework can be uniformly described and classified by the new concept of auxiliary operator. Some well-known previous uncertainty relations can be recovered by the weakest two classes of the unified and exact framework, and the deficiencies of them are, in fact, the common characteristics of the classes they belong to. The deficiencies can be completely fixed by the stronger classes in the unified framework, which means that the unified framework not only recovers the previous uncertainty relations, but also fixes the deficiencies of them. In addition to recover previous uncertainty relations, the unified framework can also be used to construct new and stronger uncertainty relations: (i) the uncertainty relations in both product form and sum form, (ii) the uncertainty relations for two and more incompatible observables, and (iii) the uncertainty relations with any tightness we need, including the uncertainty equality, which can be considered as the uncertainty relations possessing tightest lower bound.

An application has been provided to illustrate that the uncertainty relations in the stronger class, in particular, the exact Class-Cr, can be used to detect the non-Markovianity more effectively. The application indicates that the unified and exact framework not only is of fundamental interest, but also has some important applications in quantum information science.

The previous uncertainty relations are contradictory with the non-Hermitian operators, because most of the uncertainty relations will be violated when applied to the non-Hermitian operators. By the non-Hermitian extension of the commutator and anti-commutator, the unified framework can be well applied to non-Hermitian operators. Also, the equality based on the second-order origin moments provides a new interpretation of the uncertainty relation for the non-Hermitian operators, i.e., the second-order origin moments of the non-Hermitian operators cannot be arbitrarily small at the same time when they are generalized incompatible or generalized anti-incompatible with each other. The new interpretation reveals some novel quantum properties that the traditional uncertainty relation cannot do.

## Appendix

### Appendix A: The Equality Based on the Second-Order Origin Moments

Considering two arbitrary operators ${\mathscr{A}}$ and ${\mathcal B}$, it is easy to verify that the bilinear operator function $\langle {{\mathscr{A}}}^{\dagger } {\mathcal B} \rangle$ has the following property^63^:

S1 $$\langle {{\mathscr{A}}}^{\dagger } {\mathcal B} \rangle ={\langle { {\mathcal B} }^{\dagger }{\mathscr{A}}\rangle }^{\ast }.$$

Meanwhile, the operator ${\mathscr{A}}$ can be decomposed as:

S2 $${\mathscr{A}}=\frac{\langle { {\mathcal B} }^{\dagger }{\mathscr{A}}\rangle }{\langle { {\mathcal B} }^{\dagger } {\mathcal B} \rangle } {\mathcal B} +({\mathscr{A}}-\frac{\langle { {\mathcal B} }^{\dagger }{\mathscr{A}}\rangle }{\langle { {\mathcal B} }^{\dagger } {\mathcal B} \rangle } {\mathcal B} ).$$

The coefficient $\langle { {\mathcal B} }^{\dagger }{\mathscr{A}}\rangle /\langle { {\mathcal B} }^{\dagger } {\mathcal B} \rangle$ is considered to be zero hereafter when $\langle { {\mathcal B} }^{\dagger } {\mathcal B} \rangle$ happens to be zero. Taking advantage of Eqs. (S1) and (S2), one has:

S3 $$\langle {{\mathscr{A}}}^{\dagger }{\mathscr{A}}\rangle =\frac{|\langle {{\mathscr{A}}}^{\dagger } {\mathcal B} \rangle {|}^{2}}{\langle { {\mathcal B} }^{\dagger } {\mathcal B} \rangle }+\langle ({{\mathscr{A}}}^{\dagger }-\frac{\langle {{\mathscr{A}}}^{\dagger } {\mathcal B} \rangle }{\langle { {\mathcal B} }^{\dagger } {\mathcal B} \rangle }{ {\mathcal B} }^{\dagger })({\mathscr{A}}-\frac{\langle { {\mathcal B} }^{\dagger }{\mathscr{A}}\rangle }{\langle { {\mathcal B} }^{\dagger } {\mathcal B} \rangle } {\mathcal B} )\rangle .$$

Multiplying the two sides of Eq. (S3) by $\langle { {\mathcal B} }^{\dagger } {\mathcal B} \rangle$, one can obtain Eq. (1).

Example to demonstrate the importance of the remainder: Assume that the state of a qubit system is:

S4 $$\rho =(\begin{array}{ll}1/2 & 1/\sqrt{8}\\ 1/\sqrt{8} & 1/2\end{array}).$$

Taking ${\mathscr{A}}={\sigma }_{x}-{\sigma }_{x}$, and ${\mathcal B} ={\sigma }_{z}-{\sigma }_{z}$, one can obtain that Eq. (1) reduces to:

S5 $$\Delta {\sigma }_{x}^{2}\Delta {\sigma }_{z}^{2}=0+0+\frac{1}{2},$$

where the remainder becomes 1/2, and the other parts of lower bound (1), which actually constitute the lower bound of SUR, turn into 0. *σ*_*x*_ and *σ*_*z*_ are incompatible with each other. Based on the uncertainty relation, we have the variances of them, namely $\Delta {\sigma }_{x}^{2}$ and $\Delta {\sigma }_{z}^{2}$, cannot be arbitrarily small at the same time. According to Eq. (S5), we can see that the lower bound (1) is trivial in capturing the incompatibility between *σ*_*x*_ and *σ*_*z*_ in some cases, if we do not consider the remainder.

### Appendix B: Triviality Problem of Class-C0

The uncertainty relations in Class-C0 can be deduced from ${\mathbb{D}}:\ge 0$. For *N* = 2, one has:

S6 $${\mathbb{D}}=(\begin{array}{ll}\langle {\check{{A}}}_{1}^{\dagger }{\check{{A}}}_{1}\rangle  & \langle {\check{{A}}}_{1}^{\dagger }{\check{{A}}}_{2}\rangle \\ \langle {\check{{A}}}_{2}^{\dagger }{\check{{A}}}_{1}\rangle  & \langle {\check{{A}}}_{2}^{\dagger }{\check{{A}}}_{2}\rangle \end{array}):\ge 0.$$

In the finite-dimension system, assume that the state of the system happens to be an eigenstate of *A*_2_, and Eq. (S6) turns into:

S7 $${\mathbb{D}}=(\begin{array}{ll}\langle {\check{{A}}}_{1}^{\dagger }{\check{{A}}}_{1}\rangle  & 0\\ 0 & 0\end{array}):\ge 0.$$

Uncertainty relation indicates that the variances of the incompatible observables *A*_1_ and *A*_2_ cannot be arbitrarily small at the same time. That is to say, $\Delta {A}_{1}^{2} > 0$ when the state of the system happens to be the eigenstate of *A*_2_, namely $\Delta {A}_{2}^{2}=0$. However, this essence of uncertainty relation cannot be fully described by (S6). From (S7), we can only deduce that $\Delta {A}_{1}^{2}\ge 0$, instead of $\Delta {A}_{1}^{2} > 0$. Thus, (S6), so are the uncertainty relations directly deduced from it, is trivial when $\Delta {A}_{2}^{2}=0$. Similar deduction can be extended to those cases for *N* > 2.

### Appendix C: Triviality Problem can be Fixed by Introducing the Auxiliary Operator with Non-Zero Second-Order Origin Moment

Assuming an auxiliary operator ${\mathscr{O}}$, and taking advantage of Eq. (8), then we can obtain a sum form uncertainty relation for two incompatible observables:

S8 $$\Delta {A}^{2}+\Delta {B}^{2}\ge \frac{|\langle {[(\check{{A}}+{e}^{i\theta }\check{B}),{\mathscr{O}}]}_{{\mathscr{G}}}\rangle {|}^{2}+|\langle {\{(\check{{A}}+{e}^{i\theta }\check{B}),{\mathscr{O}}\}}_{{\mathscr{G}}}\rangle {|}^{2}}{4|\langle {{\mathscr{O}}}^{\dagger }{\mathscr{O}}\rangle |}-\langle {\{\check{{A}},{e}^{i\theta }\check{B}\}}_{{\mathscr{G}}}\rangle ,$$

where *θ* should be chosen to maximize the lower bound. By adjusting *θ*, the second term in lower bound (S8), namely $-\langle {\{\check{{A}},{e}^{i\theta }\check{B}\}}_{{\mathscr{G}}}\rangle$, can be guaranteed to be greater than or equal to 0. Thus, the triviality problem is fixed when the first term $(|\langle {[(\check{{A}}+{e}^{i\theta }\check{B}),{\mathscr{O}}]}_{{\mathscr{G}}}\rangle {|}^{2}+|\langle {\{(\check{{A}}+{e}^{i\theta }\check{B}),{\mathscr{O}}\}}_{{\mathscr{G}}}\rangle {|}^{2})/4|\langle {{\mathscr{O}}}^{\dagger }{\mathscr{O}}\rangle |$ is not equal to zero. The numerator of $(|\langle {[(\check{{A}}+{e}^{i\theta }\check{B}),{\mathscr{O}}]}_{{\mathscr{G}}}\rangle {|}^{2}+|\langle {\{(\check{{A}}+{e}^{i\theta }\check{B}),{\mathscr{O}}\}}_{{\mathscr{G}}}\rangle {|}^{2})/4|\langle {{\mathscr{O}}}^{\dagger }{\mathscr{O}}\rangle |$ can be considered as the lower bound of a SUR-type uncertainty relation:

S9 $$\langle {{\mathscr{A}}}^{\dagger }{\mathscr{A}}\rangle \langle { {\mathcal B} }^{\dagger } {\mathcal B} \rangle \ge \frac{1}{4}|\langle i{[{\mathscr{A}}, {\mathcal B} ]}_{{\mathscr{G}}}\rangle {|}^{2}+\frac{1}{4}|\langle {\{{\mathscr{A}}, {\mathcal B} \}}_{{\mathscr{G}}}\rangle {|}^{2},$$

where ${\mathscr{A}}=\check{{A}}+{e}^{i\theta }\check{B}$ and ${\mathcal B} ={\mathscr{O}}$. The lower bound (S9) will be equal to zero when the second-order origin moment of ${\mathcal B}$ is zero. That is to say, to ensure that the lower bound (S8) is non-trivial, the auxiliary operator ${\mathscr{O}}$ should satisfy $\langle {{\mathscr{O}}}^{\dagger }{\mathscr{O}}\rangle \ne 0$. The uncertainty relation turns into an equality $\Delta {A}^{2}+\Delta {B}^{2}=-\langle {\{\check{{A}},{e}^{i\theta }\check{B}\}}_{{\mathscr{G}}}\rangle$ when the second-order origin moment of ${\mathscr{A}}$ is equal to zero. Since $\Delta {A}^{2}+\Delta {B}^{2}$ is never equal to zero when there does not exist common eigenstate between $A$ and $B$, the lower bound of uncertainty equality is guranteed to be non-trivial. Meanwhile, we should mention that the lower bound (S9) is zero in some occasional cases, even when $\langle {{\mathscr{A}}}^{\dagger }{\mathscr{A}}\rangle \ne 0$ and $\langle { {\mathcal B} }^{\dagger } {\mathcal B} \rangle \ne 0$. In order to avoid such an occasional case, we should choose ${\mathscr{O}}$ that does not satisfy the condition $\langle \check{{A}}\check{B}\rangle \equiv \langle {{\mathscr{O}}}^{\dagger }(\check{{A}}+{e}^{i\theta }\check{B})\rangle \equiv 0$. Using the arbitrariness of *θ*, the condition can also be written as $\langle \check{{A}}\check{B}\rangle \equiv \langle {{\mathscr{O}}}^{\dagger }\check{{A}}\rangle \equiv \langle {{\mathscr{O}}}^{\dagger }\check{B}\rangle \equiv 0$. To avoid satisfying such a condition is always possible, and the triviality problem can be fixed by almost any choice of ${\mathscr{O}}$. The uncertainty relation (S8) turns to (4) when ${\mathscr{O}}=|{\psi }^{\perp }\rangle \langle \psi |$ and $\theta =\pm \pi /2$. Correspondingly, the condition $\langle \check{{A}}\check{B}\rangle \equiv \langle {{\mathscr{O}}}^{\dagger }(\check{{A}}+{e}^{i\theta }\check{B})\rangle \equiv 0$ becomes $\langle {\psi }^{\perp }|A\pm iB|\psi \rangle \equiv 0$. That is to say, to fix the triviality problem by the uncertainty relation (4), we should choose $|{\psi }^{\perp }\rangle$ to avoid $\langle {\psi }^{\perp }|A\pm iB|\psi \rangle \equiv 0$.

As for uncertainty relation (11), which is obtained by introducing two generalized incompatible or incompatible auxiliary operators, to avoid the occasional cases mentioned above, we should choose ${\mathcal R}$ and ${\mathscr{S}}$ that can avoid $\langle \check{{A}}\check{B}\rangle \equiv \langle { {\mathcal R} }^{\dagger }\check{{A}}\rangle \equiv \langle { {\mathcal R} }^{\dagger }\check{B}\rangle \equiv \langle {{\mathscr{S}}}^{\dagger }\check{{A}}\rangle \equiv \langle {{\mathscr{S}}}^{\dagger }\check{B}\rangle \equiv 0$.

### Appendix D: Exact Class-Cr

Based on Eq. (12), one has:

S10 $$\langle { {\mathcal F} }_{1}^{\dagger }{ {\mathcal F} }_{1}\rangle =\mathop{\sum }\limits_{k=1}^{m}\,{ {\mathcal L} }_{k}+\langle { {\mathcal F} }_{m+1}^{\dagger }{ {\mathcal F} }_{m+1}\rangle ,$$

where ${ {\mathcal F} }_{m+1}={ {\mathcal F} }_{1}-\mathop{\sum }\limits_{k=1}^{m}\,\langle {{\mathscr{O}}}_{k}^{\dagger }{ {\mathcal F} }_{k}\rangle {{\mathscr{O}}}_{k}/|\langle {{\mathscr{O}}}_{k}^{\dagger }{{\mathscr{O}}}_{k}\rangle |$ and ${{\mathscr{O}}}_{k}$ represents an arbitrary operator, with $k=1,\,2,\,\cdots ,\,m.$ The operator ${{\mathscr{O}}}_{k}$ can make up a set $\{{{\mathscr{O}}}_{1},\,{{\mathscr{O}}}_{2},\,\cdots ,\,{{\mathscr{O}}}_{m}\}$. In the following, we assume that the elements in the set $\{{{\mathscr{O}}}_{1},\,{{\mathscr{O}}}_{2},\,\cdots ,\,{{\mathscr{O}}}_{k}\}$ satisfy the conditions that $\langle {{\mathscr{O}}}_{k}^{\dagger }{{\mathscr{O}}}_{l}\rangle =\langle {{\mathscr{O}}}_{k}^{\dagger }{{\mathscr{O}}}_{l}\rangle {\delta }_{kl}$ and $\langle {{\mathscr{O}}}_{k}^{\dagger }{{\mathscr{O}}}_{k}\rangle \ne 0$. The set can be obtained by making a Schmidt transformation^65^ on the basic vectors of the matrix space^66^ and the calculation process will be presented in Appendix E. Then, the term ${ {\mathcal L} }_{k}$ in Eq. (S10) is simplified as ${ {\mathcal L} }_{k}(|\langle {[{ {\mathcal F} }_{1},{{\mathscr{O}}}_{k}]}_{{\mathscr{G}}}\rangle {|}^{2}+|\langle {\{{ {\mathcal F} }_{1},{{\mathscr{O}}}_{k}\}}_{{\mathscr{G}}}\rangle {|}^{2})/4|\langle {{\mathscr{O}}}_{k}^{\dagger }{{\mathscr{O}}}_{k}\rangle |$, and ${ {\mathcal F} }_{m+1}$ turns into ${ {\mathcal F} }_{m+1}={ {\mathcal F} }_{1}-\mathop{\sum }\limits_{k=1}^{m}\,\langle {{\mathscr{O}}}_{k}^{\dagger }{ {\mathcal F} }_{1}\rangle {{\mathscr{O}}}_{k}/|\langle {{\mathscr{O}}}_{k}^{\dagger }{{\mathscr{O}}}_{k}\rangle |$.

In the *d*^2^-dimension matrix space, an arbitrary matrix can be linearly expressed as a composition of the basic vectors $\{{M}_{1},{M}_{2},\,\cdots ,\,{M}_{{d}^{2}}\}$ of the matrix space^43,65^. Assuming that ${{\mathscr{O}}}_{k}=\mathop{\sum }\limits_{j=1}^{{d}^{2}}\,{x}_{k}^{j}{M}_{j}$, one has:

S11 $$\langle {{\mathscr{O}}}_{k}^{\dagger }{{\mathscr{O}}}_{l}\rangle ={X}_{k}^{\dagger }{\mathbb{M}}{X}_{l},$$

where ${X}_{k}={({x}_{k}^{1},{x}_{k}^{2},\cdots ,{x}_{k}^{{d}^{2}})}^{T}$ and the Metric matrix ${\mathbb{M}}$ for the bilinear function $\langle {{\mathscr{A}}}^{\dagger } {\mathcal B} \rangle$ is^65^:

S12 $${\mathbb{M}}=(\begin{array}{cccc}\langle {M}_{1}^{\dagger }{M}_{1}\rangle  & \langle {M}_{1}^{\dagger }{M}_{2}\rangle  & \cdots  & \langle {M}_{1}^{\dagger }{M}_{{d}^{2}}\rangle \\ \langle {M}_{2}^{\dagger }{M}_{1}\rangle  & \langle {M}_{2}^{\dagger }{M}_{2}\rangle  & \cdots  & \langle {M}_{2}^{\dagger }{M}_{{d}^{2}}\rangle \\ \vdots  & \vdots  & \ddots  & \vdots \\ \langle {M}_{{d}^{2}}^{\dagger }{M}_{1}\rangle  & \langle {M}_{{d}^{2}}^{\dagger }{M}_{2}\rangle  & \cdots  & \langle {M}_{{d}^{2}}^{\dagger }{M}_{{d}^{2}}\rangle \end{array})$$

According to (S1), the Metric matrix ${\mathbb{M}}$ is Hermitian, which means that ${\mathbb{M}}$ can be transformed into a diagonal matrix by a unitary transformation. Thus the number of ${{\mathscr{O}}}_{k}$ satisfying the conditions that $\langle {{\mathscr{O}}}_{k}^{\dagger }{{\mathscr{O}}}_{l}\rangle =\langle {{\mathscr{O}}}_{k}^{\dagger }{{\mathscr{O}}}_{l}\rangle {\delta }_{kl}$ and $\langle {{\mathscr{O}}}_{k}^{\dagger }{{\mathscr{O}}}_{k}\rangle \ne 0$ is exactly equal to *r*, where $r\le {d}^{2}$ is the rank of the Metric matrix ${\mathbb{M}}$, and depends only on the state of the system. Meanwhile, we have *r* is less than *d* for the *d*-dimension pure state $|\psi \rangle$, because we cannot find an operator ${{\mathscr{O}}}_{d+1}$ which satisfies $\langle {{\mathscr{O}}}_{1}^{\dagger }{{\mathscr{O}}}_{d+1}\rangle =\langle {{\mathscr{O}}}_{2}^{\dagger }{{\mathscr{O}}}_{d+1}\rangle =\langle {{\mathscr{O}}}_{3}^{\dagger }{{\mathscr{O}}}_{d+1}\rangle =\cdots =\langle {{\mathscr{O}}}_{d}^{\dagger }{{\mathscr{O}}}_{d+1}\rangle =0$ and $\langle {{\mathscr{O}}}_{d+1}^{\dagger }{{\mathscr{O}}}_{d+1}\rangle \ne 0$ when taking ${{\mathscr{O}}}_{1}=|\psi \rangle \langle \psi |,{{\mathscr{O}}}_{2}=|{\psi }_{1}^{\perp }\rangle \langle \psi |,\ldots ,{{\mathscr{O}}}_{d}=|{\psi }_{d-1}^{\perp }\rangle \langle \psi |$ with $|{\psi }_{i}^{\perp }\rangle$ being the state orthogonal to $|\psi \rangle$ and $\langle {\psi }_{i}^{\perp }|{\psi }_{j}^{\perp }\rangle ={\delta }_{ij}$. It should mention that *r* will become infinite when considering the infinite-dimensional system.

Based on the analysis above, one can obtain $$\langle { {\mathcal F} }_{r+1}^{\dagger }{ {\mathcal F} }_{r+1}\rangle =0$$ when $$\langle {{\mathscr{O}}}_{1}^{\dagger }{ {\mathcal F} }_{r+1}\rangle =\langle {{\mathscr{O}}}_{2}^{\dagger }{ {\mathcal F} }_{r+1}\rangle =\langle {{\mathscr{O}}}_{3}^{\dagger }{ {\mathcal F} }_{r+1}\rangle =\cdots =$$$$\langle {{\mathscr{O}}}_{r}^{\dagger }{ {\mathcal F} }_{r+1}\rangle =0$$. Obviously, $$\langle {{\mathscr{O}}}_{m}^{\dagger }{ {\mathcal F} }_{r+1}\rangle =\langle {{\mathscr{O}}}_{m}^{\dagger }{ {\mathcal F} }_{1}\rangle -\langle {{\mathscr{O}}}_{m}^{\dagger }{ {\mathcal F} }_{m}\rangle =0$$ with $$m\in \{1,2,\ldots ,r\}$$, and thus we can deduce $$\langle { {\mathcal F} }_{r+1}^{\dagger }{ {\mathcal F} }_{r+1}\rangle =0$$. Then, Eq. (S10) turns into:

S13 $$\langle { {\mathcal F} }_{1}^{\dagger }{ {\mathcal F} }_{1}\rangle =\mathop{\sum }\limits_{k=1}^{r}\,{ {\mathcal L} }_{k}.$$

Taking ${ {\mathcal F} }_{1}=\mathop{\sum }\limits_{m=1}^{N}\,{x}_{m}{\check{{A}}}_{m}$ with *x*_*m*_ ∈ *C* and *A*_*m*_ being an arbitrary operator, one can obtain:

S14 $${X}^{\dagger }.{\mathbb{D}}.X={X}^{\dagger }.(\mathop{\sum }\limits_{k=1}^{r}\,{{\mathbb{V}}}_{k}).X,$$

where ${\mathbb{D}}$ is a *N* × *N* dimension matrix with the elements ${\mathbb{D}}(m,n)=\langle {\check{{A}}}_{m}^{\dagger }{\check{{A}}}_{n}\rangle$, ${{\mathbb{V}}}_{k}$ is the *N* × *N* dimension positive semidefinite matrix with the elements ${{\mathbb{V}}}_{k}(m,n)=\langle {\check{{A}}}_{m}^{\dagger }{{\mathscr{O}}}_{k}\rangle \langle {{\mathscr{O}}}_{k}^{\dagger }{\check{{A}}}_{n}\rangle /|\langle {{\mathscr{O}}}_{k}^{\dagger }{{\mathscr{O}}}_{k}\rangle |$, and $X={\{{x}_{1},{x}_{2},\cdots ,{x}_{N}\}}^{T}$. Making use of the random of *X*, one can obtain:

S15 $${\mathbb{D}}=\mathop{\sum }\limits_{k=1}^{r}\,{{\mathbb{V}}}_{k}.$$

The deduction above shows that the Eq. (S13) can be reformed as ${\mathbb{D}}=\mathop{\sum }\limits_{k=1}^{r}\,{{\mathbb{V}}}_{k}$. Similarly, one can also deduce that Eq. (12) can be reformed as ${\mathbb{D}}:\ge \mathop{\sum }\limits_{k=1}^{m}\,{{\mathbb{V}}}_{k}$ when the auxiliary operators satisfy the conditions that $\langle {{\mathscr{O}}}_{k}^{\dagger }{{\mathscr{O}}}_{l}\rangle =\langle {{\mathscr{O}}}_{k}^{\dagger }{{\mathscr{O}}}_{l}\rangle {\delta }_{kl}$ and $\langle {{\mathscr{O}}}_{k}^{\dagger }{{\mathscr{O}}}_{k}\rangle \ne 0$.

### Appendix E: Schmidt Transformation Process

In order to obtain the set $\{{{\mathscr{O}}}_{1},{{\mathscr{O}}}_{2},\cdots ,{{\mathscr{O}}}_{r}\}$ that satisfies $\langle {{\mathscr{O}}}_{k}^{\dagger }{{\mathscr{O}}}_{l}\rangle =\langle {{\mathscr{O}}}_{k}^{\dagger }{{\mathscr{O}}}_{l}\rangle {\delta }_{kl}$ and $\langle {{\mathscr{O}}}_{k}^{\dagger }{{\mathscr{O}}}_{k}\rangle \ne 0$, we can make a Schmidt transform on the basic vectors $\{{M}_{1},{M}_{2},\,\cdots ,\,{M}_{{d}^{2}}\}$ of the matrix space^65,66^:

S16 $$\begin{array}{ccc}{{\mathscr{O}}^{\prime} }_{1} & = & {M}_{1},\\ {{\mathscr{O}}^{\prime} }_{2} & = & {M}_{2}-\frac{\langle {{\mathscr{O}}^{\prime} }_{1}^{\dagger }{M}_{2}\rangle }{\langle {{\mathscr{O}}^{\prime} }_{1}^{\dagger }{{\mathscr{O}}^{\prime} }_{1}\rangle }{{\mathscr{O}}^{\prime} }_{1},\\  & \vdots  & \\ {{\mathscr{O}}^{\prime} }_{{d}^{2}} & = & {M}_{{d}^{2}}-\frac{\langle {{\mathscr{O}}^{\prime} }_{1}^{\dagger }{M}_{{d}^{2}}\rangle }{\langle {{\mathscr{O}}^{\prime} }_{1}^{\dagger }{{\mathscr{O}}^{\prime} }_{1}\rangle }{{\mathscr{O}}^{\prime} }_{1}-\cdots -\frac{\langle {{\mathscr{O}}^{\prime} }_{{d}^{2}-1}^{\dagger }{M}_{{d}^{2}}\rangle }{\langle {{\mathscr{O}}^{\prime} }_{{d}^{2}-1}^{\dagger }{{\mathscr{O}}^{\prime} }_{{d}^{2}-1}\rangle }{{\mathscr{O}}^{\prime} }_{{d}^{2}-1}.\end{array}$$

Here we take $\langle {{\mathscr{O}}}_{m}^{^{\prime} \dagger }{M}_{n}\rangle /\langle {{\mathscr{O}}}_{m}^{^{\prime} \dagger }{{\mathscr{O}}}_{m}^{^{\prime} }\rangle =0$ when $\langle {{\mathscr{O}}}_{m}^{^{\prime} \dagger }{M}_{n}\rangle =0$. Obviously, the set $\{{{\mathscr{O}}}_{1}^{^{\prime} },{{\mathscr{O}}}_{2}^{^{\prime} },\,\cdots ,\,{{\mathscr{O}}}_{{d}^{2}}^{^{\prime} }\}$ satisfies $\langle {{\mathscr{O}}}_{k}^{^{\prime} \dagger }{{\mathscr{O}}}_{l}^{^{\prime} }\rangle =\langle {{\mathscr{O}}}_{k}^{^{\prime} \dagger }{{\mathscr{O}}}_{l}^{^{\prime} }\rangle {\delta }_{kl}$. Dropping out ${{\mathscr{O}}}_{k}^{^{\prime} }$ when $\langle {{\mathscr{O}}}_{k}^{^{\prime} \dagger }{{\mathscr{O}}}_{k}^{^{\prime} }\rangle =0$, one can obtain the operator set $\{{{\mathscr{O}}}_{1},{{\mathscr{O}}}_{2},\cdots ,{{\mathscr{O}}}_{r}\}$. As for the Figs. 1 and 3, the basic vectors of the matrix space is taken as *E*_*ij*_ with the elements ${E}_{ij}(m,n)={\delta }_{im}{\delta }_{jn}$.

Here, we should mention that the auxiliary operators involved should satisfy $\langle {{\mathscr{O}}}_{k}^{\dagger }{{\mathscr{O}}}_{l}\rangle =\langle {{\mathscr{O}}}_{k}^{\dagger }{{\mathscr{O}}}_{l}\rangle {\delta }_{kl}$ and $\langle {{\mathscr{O}}}_{k}^{\dagger }{{\mathscr{O}}}_{k}\rangle \ne 0$ only in the process to construct uncertainty equality. As for the construction of uncertainty inequalities, such as the uncertainty relations in Class-C1, Class-C2, and Class-Cm, the auxiliary operators introduced is unrestricted.

### Appendix F: Introduction of Choi State

Consider an arbitrary evolution ${ {\mathcal E} }_{(t,t+\varepsilon )}$:

S17 $${ {\mathcal E} }_{(t,t+\varepsilon )}:\rho (t)\to \rho (t+\varepsilon ),\,{ {\mathcal H} }_{1}\to { {\mathcal H} }_{1},$$

where *ρ*(*t*) and *ρ*(*t* + *ε*) are the quantum state in the Hilbert space ${ {\mathcal H} }_{1}$, with *ε* being a arbitrary time interval. Assume another Hilbert space ${ {\mathcal H} }_{2}$, and let $|\psi \rangle$ represents the maximally entangled state of the Hilbert space ${ {\mathcal H} }_{1}\otimes { {\mathcal H} }_{2}$. Then, the Choi state of the evolution ${ {\mathcal E} }_{(t,t+\varepsilon )}$ is defined as^67–70^:

S18 $${\rho }_{Choi}=[{\mathbb{I}}\otimes { {\mathcal E} }_{(t,t+\varepsilon )}](|\psi \rangle \langle \psi |),$$

where ${\mathbb{I}}$ is the identity evolution of ${ {\mathcal H} }_{2}$. Assume the two Hilbert space ${ {\mathcal H} }_{1}$ and ${ {\mathcal H} }_{2}$ are the same, then the Choi state of evolution (14) is obtained:

S19 $${\rho }_{Choi}=(\begin{array}{llll}\frac{1}{2} & 0 & 0 & \frac{1}{2}{e}^{\frac{-4{\gamma }_{0}\varepsilon \lambda }{\lambda +gcoth(tg/2)}}\\ 0 & 0 & 0 & 0\\ 0 & 0 & 0 & 0\\ \frac{1}{2}{e}^{\frac{-4{\gamma }_{0}\varepsilon \lambda }{\lambda +gcoth(tg/2)}} & 0 & 0 & \frac{1}{2}\end{array}),$$

where the maximally entangled state is $|\psi \rangle =1/\sqrt{2}{(|0\rangle }_{1}|0{\rangle }_{2}+|1{\rangle }_{1}|1{\rangle }_{2})$, with ${\{|0\rangle }_{i},|1{\rangle }_{i}\}$ being the orthogonal basis of Hilbert space ${ {\mathcal H} }_{i}$.
